# School mental health best practices institute; capacity building of teachers in mental health literacy in Pakistan

**DOI:** 10.1192/bjo.2021.407

**Published:** 2021-06-18

**Authors:** Maria Khan, Aisha Sanober Chachar, Wamiq Ali, Ayesha Mian

**Affiliations:** 1 The Aga Khan University Hospital; 2 Alleviate Addiction Suffering Trust; 3 Dow University of Health Sciences

## Abstract

**Aims:**

Mental health disorders in children are largely unrecognized in low- and middle-income countries like Pakistan. Teachers, due to their interactions and time spent with children, are important elements in promoting child mental health. Despite this, little importance is given to school mental health (SMH) in the country, and teachers’ training in SMH is almost non-existent. With less than ten child and adolescent psychiatrists, recruiting teachers is vital to provide mental health care to children and adolescents, the majority of the country's population. This study aims to evaluate the effectiveness of a SMH training intervention for teachers in Pakistan.

**Method:**

A 3-day training intervention was planned for school teachers in collaboration with International Association of Child and Adolescent Psychiatry and Allied Professions. The School Mental Health curriculum by the World Health Organization and Stan Kutcher's Mental Health Literature were adapted after literature review and discussions with experts, and administered as Blended Learning. The intervention was evaluated using pre-workshop, post-workshop and overall feedback surveys. SPSS 25.0 (IBM Corp., Armonk, N.Y., USA) software was used for descriptive analysis. For open ended questions, central themes were identified, tabulated, and analyzed descriptively.

**Result:**

A total of 63 participants registered for the workshop. The participants’ mean age was 36.0 years, with 86% women and 14% men. Participants were mostly teachers, however, principals, administrators and counsellors also attended. Participants’ reasons for attending were that they wanted to ‘increase their knowledge’ and learn ‘practical management’ of mental health issues. When asked about student wellbeing, 43% participants said it was a neglected area. Overall, 86.9% of participants felt the objectives were met well or very well and 87.61% stated there was adequate time for discussion. In addition, 90.47% participants responded that facilitators explained concepts well and 94.39% said facilitators answered questions well. Half of all Blended Learning activities were viewed by more than 50% of participants. Activity views decreased by 63.41% from the pre-workshop activities to day 3 activities. Improvements suggested by participants included taking a more problem-solving approach and focusing on the local context.

**Conclusion:**

Evidence-based SMH interventions that train teachers are much-needed in the local resource-constrained settings. This intervention met its objectives effectively, however, Blended Learning was not well-received. We have studied learning analytics and identified the potential learner's profile of teachers as students. Adult learning principles should be implemented in future endeavours. This is a flagship project for future international collaborations between mental health professionals for cross-cultural knowledge exchange.

